# Post-Prandial Cognitive and Blood Pressure Effects of a DHA-Rich Omega-3 Powder in Middle-Aged Males: A Pilot Study

**DOI:** 10.3390/nu15092198

**Published:** 2023-05-05

**Authors:** Andrew Pipingas, Jeffery Michael Reddan, Sarah Gauci, Lauren M. Young, Greg Kennedy, Renee Rowsell, Rebecca King, Sam Spiteri, Anne Marie Minihane, Andrew Scholey

**Affiliations:** 1 Centre for Mental Health and Brain Sciences, Swinburne University of Technology, Hawthorn, VIC 3122, Australia; jreddan@swin.edu.au (J.M.R.); lauren.young@monash.edu (L.M.Y.); gikennedy@swin.edu.au (G.K.); reneecastell17@gmail.com (R.R.); rking@swin.edu.au (R.K.); sspiteri@swin.edu.au (S.S.); andrew@scholeylab.com (A.S.); 2 Food & Mood Centre, The Institute for Mental and Physical Health and Clinical Translation (IMPACT), School of Medicine, Deakin University, Geelong, VIC 3220, Australia; sarah.gauci@deakin.edu.au; 3 Norwich Medical School, University of East Anglia, Norwich, NR4 7TJ, UK; a.minihane@uea.ac.uk; 4 Department of Nutrition, Dietetics and Food, Monash University, Notting Hill, VIC 3168, Australia

**Keywords:** omega-3 PUFA, DHA, nootropic, middle-aged, acute, cognition, blood pressure

## Abstract

The use of omega-3 polyunsaturated fatty acid (ω-3 PUFA) supplements is increasingly common among middle-aged and older adults. Users of ω-3 PUFA supplements often report using such supplements to support cognitive health, despite mixed findings reported within the ω-3 PUFA literature. To date, very few studies have explored cognitive effects in distinctly middle-aged (40 to 60 years) adults, and none have examined the acute effects (in the hours following a single dose) on cognitive performance. The current study evaluated whether a single dose of ω-3 PUFA (4020 mg docosahexaenoic acid and 720 mg eicosapentaenoic acid) influences cognitive performance and cardiovascular function in middle-aged males. Cognitive performance and cardiovascular function were assessed before and 3.5–4 h after consumption of a high dose of ω-3 PUFA (DHA + EPA) or placebo, incorporated into a standardized meal (i.e., single serve of Greek yogurt). In this study of middle-aged males, no significant differential treatment effects were observed for cognitive performance. However, a significant reduction in aortic systolic blood pressure (pre-dose to post-dose) was apparent following consumption of the ω-3 PUFA (DHA + EPA) treatment (mean difference = −4.11 mmHg, *p* = 0.004) but not placebo (mean difference = −1.39 mmHg, *p* = 0.122). Future replication in a sample comprising females, as well as patients with hypertension, is merited.

## 1. Introduction

The use of complimentary and natural medicines to support health is a relatively common occurrence, even in industrialized nations such as Australia, the United States, and the United Kingdom [1]. One recent report estimated that Australians spend approximately AUD 2.9 billion on complimentary medicines annually [2], the most common medicines being vitamin and mineral supplements [2,3]. Moreover, the self-reported use of dietary supplements has been increasing over time in young, middle-aged, and older populations [4,5,6]. A survey of North American supplement users indicated that people often utilize dietary supplements as a means of supporting cognitive health when experiencing ‘cognitive problems’, though prophylactic use to support cognitive function in the absence of specific impairment is also relatively common [7]. Omega-3 polyunsaturated fatty acids (ω-3 PUFA)—specifically, docosahexaenoic acid (DHA) and eicosapentaenoic acid (EPA)—are perceived as beneficial to cognitive function [8], with some reports indicating that they are perhaps the most common non-vitamin product utilized to support cognitive health [7].

Preclinical and prospective cohort studies have consistently reported that a high DHA and EPA intake or status is associated with a reduced risk of dementia, cognitive decline, and larger brain volumes, with several mechanistic pathways having been identified [9,10]. However, observational studies cannot determine causality and clinical trials have reported both positive but largely null effects of ω-3 PUFA (DHA + EPA) upon cognitive function [9,11]. Subsequently, there is a lack of consensus and clarity regarding the determinants of better cognitive outcomes typically associated with increased DHA and EPA intake or status. Thus, more well-designed clinical trials are required. 

The effects of ω-3 PUFA (typically DHA + EPA) supplementation on cognitive function has been examined in children, adolescents, and adults of all ages [11,12]. In their scoping review, Andriambelo and colleagues [11] consider 24 studies in adults aged 20 to 60 years. Most of the included studies with healthy cohorts examined cognitive effects in younger adults (i.e., under 35 years), with four including both young and middle-aged adults. Two of these studies [13,14] report positive effects of ω-3 PUFA (DHA + EPA) supplementation on cognitive function in adults aged 25 to 49 and 18 to 45 years, respectively. Another [15] reported improved cognitive performance following ω-3 PUFA (DHA + EPA) supplementation, but only in adults (30 to 54 years) with low red blood cell DHA at baseline. Overall, there is a paucity of studies exploring whether cognitive benefits are evident following ω-3 PUFA (DHA + EPA) supplementation in distinctly middle-aged adults (≈40 to 60 years). Considering that neuropathologies associated with dementia appear to begin in middle-age, several decades prior to a clinical diagnosis, middle-age may represent a ‘window of opportunity’ for therapeutic interventions such as ω-3 PUFA (DHA + EPA) supplementation to positively impact neurocognitive health in later life. Moreover, it appears that the cognitive effects have only been examined following chronic ω-3 PUFA supplementation (i.e., weeks, months, or years). It is possible that high intake of ω-3 PUFA (DHA + EPA) may also provide acute, post-prandial effects such as improved cognitive performance in the hours following ingestion.

The acute effects of a single dose of other nutrients on cognition have been examined previously, a pertinent example being cocoa flavanols. A single dose of cocoa flavanols have been shown to enhance executive function [16,17], working memory [18,19], spatial attention [20], and verbal episodic memory [21] performance in young and middle-aged adults within 1.5 to 3.5 h of consumption. Cocoa flavanols have also been shown to have acute benefits on endothelial function [22,23,24,25]. Data from a recent study by Gratton and colleagues [26] indicated that endothelial function and blood oxygenation (assessed using functional near infra-red spectroscopy; fNIRS) reflecting cerebrovascular reactivity were significantly greater during a hypercapnia challenge in adults who had consumed a high cocoa flavanol drink compared to a low flavanol drink. The authors also report that compared with low flavanol intake, high flavanol intake led to better performance during the incongruent Stroop task (a measure of selective attention, response inhibition, and cognitive control). Importantly, Gratton and colleagues [26] demonstrated the coupling of these two effects, with participants demonstrating increased cerebrovascular reactivity (following consumption of the high flavanol drink) also being the ones demonstrating greater cognitive performance during high demand testing.

Dietary supplementation with ω-3 PUFA (specifically DHA and/or EPA) over periods ranging from 14 days to 1 year has also been shown to improve endothelial function [27]. Moreover, there is evidence that ω-3 PUFA consumption may induce acute improvements in endothelial function and/or mitigate poorer endothelial function following a high fat meal [28,29,30,31]. Furthermore, improvements in vascular reactivity four hours after ω-3 PUFA intake have been reported in young adults (mean age = 30 years), but not older adults (mean age = 63 years), following a high-fat meal [32]. Administration of a high dose of ω-3 PUFA has also been shown to facilitate acute improvements in vascular tone (i.e., arterial stiffness) compared to placebo [33,34].

To our knowledge, no study has investigated acute cognitive effects after ingesting a single, high dose of ω-3 PUFA (DHA + EPA). However, as ω-3 PUFA consumption appears to acutely effect mechanisms such as endothelial function, similar to cocoa flavanols, there is the possibility that a single high dose of ω-3 PUFA (DHA + EPA) may acutely benefit cognitive performance. As such, the primary aim of the current study was to examine whether a single high dose of ω-3 PUFA (DHA + EPA) incorporated into a meal (i.e., a single serving of Greek yogurt), would provide acute benefits upon cognitive performance in middle-aged adults. We also sought to determine whether ω-3 PUFA (DHA + EPA) consumption would acutely benefit brachial and aortic measures of cardiovascular function and vascular tone.

## 2. Materials and Methods

The trial was a randomized, double-blind, placebo-controlled, crossover study. All participants provided written informed consent before participation in the study which was approved by the Swinburne University Human Research Ethics Committee (2018/160). All procedures were conducted in accordance with the International Council for Harmonization Guideline for Good Clinical Practice (ICH GCP). The trial was registered with the Australian and New Zealand Clinical Trials Registry (ACTRN12618001160224).

### 2.1. Participants

The trial was conducted from July 2018 to April 2021. Males aged between 40 and 60 years were recruited from a confidential database of previous participants who were interested in future nutrition studies, as well as by distributing flyers around the university and surrounding community and utilizing social media advertising. In this pilot study, we limited recruitment to males to achieve a more homogenous study cohort. Sex differences in cognitive performance have been reported previously [35,36], which may be due, in part, to the effects of different sex hormones [35]. A male-only cohort also avoids cognitive function being influenced by menopause transition [37] likely to occur in female participants within the target age range. Participants were excluded if they had cardiovascular disease, a brachial systolic blood pressure >160 mmHg or diastolic blood pressure > 90 mmHg, type I diabetes or pharmaceutically managed type II diabetes, recent (within 6 months) head trauma, dementia, cognitive impairment (MMSE < 24; [38]), a history of a neurological (e.g., stroke) or psychological (i.e., depression, anxiety in the previous two years) condition, or any gastrointestinal, endocrine, or bleeding disorder. Further exclusions included current smokers taking anti-hypertensive or cholesterol lowering medications, or current users of dietary supplements with the potential to influence cognitive performance (<4 weeks prior to enrolment). Prospective participants were also required to consume no more than one serving of fish/seafood per week.

**Figure 1:**
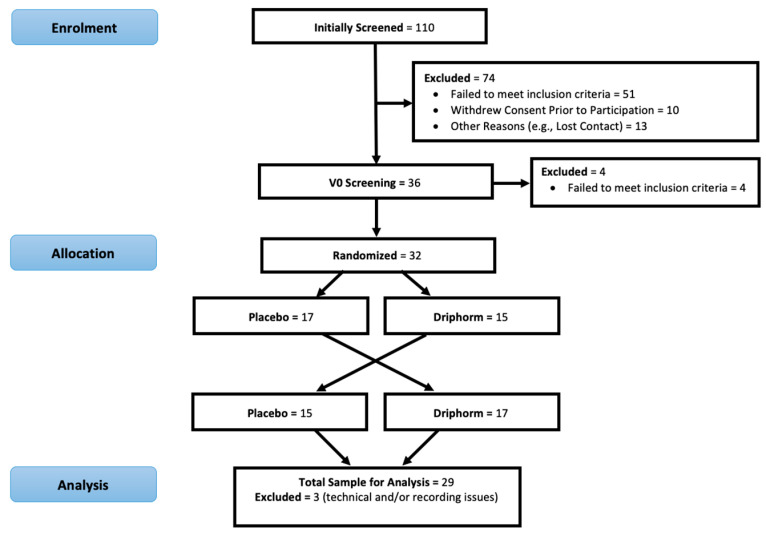
Overview of trial design, participant recruitment, and retention.

A total of 110 participants underwent telephone screening, with 36 completing in person screening at the Centre for Mental Health and Brain Sciences (Swinburne University of Technology, Melbourne). Ultimately, 32 participants were randomized and completed the trial. Furthermore, 3 participants had missing data due to technical and/or recording issues, while 29 participants finished the trial with complete data. See Figure 1 for an overview of the crossover design of the trial as well as participant recruitment and retention.

### 2.2. Experimental Treatments

The experimental treatment was microencapsulated tuna oil powder (Driphorm^®^ HiDHA^®^ 360), manufactured and supplied in kind by Nu-Mega Ingredients Limited (Altona North, Victoria, Australia), in which 12 g of this powder as a serving size incorporated a dose of 4.74 g ω-3 PUFA (4020 mg DHA and 720 mg EPA). The tuna oil powder also contained sodium caseinate, dextrose monohydrate, dried glucose syrup, sodium ascorbate, mixed natural tocopherols, and ascorbyl palmitate. The placebo was a spray dried powder matched in appearance to the active treatment. The composition of the placebo powder was identical to the active treatment except it contained sunflower oil instead of ω-3 PUFA (DHA + EPA).

Prior to administration, either treatment was combined with a single serving (140 g) of vanilla-flavored Greek yogurt, which was then mixed thoroughly to ensure textural consistency. A single drop of anchovy oil was also added to the placebo to facilitate a similar taste and smell as the experimental treatment. In order to maintain double blinding, the treatment was prepared by researchers who were not involved in either testing or analysis. Participants were required to completely consume the study treatments within 10 min.

### 2.3. Cognitive Assessments

#### Swinburne University Computerized Cognitive Assessment Battery (SUCCAB)

Cognition was assessed using the SUCCAB [39], a battery of eight computerized cognitive tasks including simple reaction time (SRT), choice reaction time (CRT), immediate (IRM) and delayed recognition memory (DRM), congruent (CS) and incongruent stroop (ICS), spatial working memory (SWM), and contextual recognition memory (CRM). Performance on these tasks has been shown to negatively correlate with age [39], and be modified by nutritional interventions [40,41]. The following is a brief description of each SUCCAB task.

##### Simple Reaction Time (SRT)

In this task, a white square was displayed on the computer monitor for 750 milliseconds (ms). Participants responded by pressing the ‘yes’ button on the response box as quickly as possible each time the square was presented.

##### Choice Reaction Time (CRT)

Participants are shown either a blue triangle or a red square on the computer monitor for 750 ms. Participants responded each time the triangle or square was presented by pressing the colored button on the response box matching the stimulus (e.g., shown blue triangle, press blue button).

##### Immediate Recognition Memory (IRM)

This task involved a memory encoding phase and a memory recognition phase. During the memory encoding phase, a series of 30 abstract images were presented on the computer monitor, 1 at a time for 3 seconds each. Participants were instructed that their memory will be tested on these images immediately after they had viewed all the images and again in approximately 20 min time (see DRM). During the recognition phase, a second series of 30 images were shown—15 images were identical to those presented during encoding, whilst the remaining 15 images were new. Participants pressed the ‘yes’ button on the response box if they recognized the displayed image as one presented during the encoding phase. Alternatively, participants pressed the ‘no’ button if they had not seen the image during the encoding phase.

##### Congruent Stroop (CS)

The words red, blue, green, or yellow were presented on the computer monitor with the words being written in the matching (i.e., congruent) ink (e.g., ‘RED’ was written in red ink). Participants responded to each word by pressing the colored button on the response box that matched the color of the stimulus word (e.g., shown the word RED, press the red button).

##### Incongruent Stroop

As with the CS task, participants were shown the words red, blue, green, or yellow on the computer monitor. However, this time the word and the ink color never matched (e.g., ‘RED’ was never in red ink, but could be written in green/yellow/blue ink). Participants were required to ignore the written word and press the colored button on the response box that matched the color of the ink the stimulus word was written in.

##### Spatial Working Memory (SWM)

During the SWM task, participants are shown 4 × 4 grid on the computer monitor for approximately three seconds with six grid locations being simultaneously filled with white squares (six white squares for six locations to be memorized; memory encoding phase). Afterwards, the white squares were removed, leaving the grid empty for a period of approximately two seconds (memory maintenance phase). A single white square was then shown in one of the grid positions for a period of approximately two seconds (memory recognition phase). Participants pressed the ‘yes’ button on the response box if the solitary white square occupied a grid space that was initially filled during the encoding phase (i.e., when the six original white squares were shown). Alternatively, participants pressed the ‘no’ button if that grid space was not initially filled during the encoding phase. The memory retrieval phase of each trial involved participants responding to four solitary white square probes, after which the encoding phase of the next trial would begin (following an inter-trial interval of approximately two seconds). A total of 14 stimulus trials were completed during this task.

##### Contextual Recognition Memory (CRM)

The CRM task involved distinct memory encoding and recognition phases. During the memory encoding phase, a series of 20 pictures of everyday objects were individually presented at the top, bottom, left, or right side of the computer monitor for a period of four seconds each. During the memory recognition phase, the same images were presented within the center of the computer monitor. Following the presentation of each picture, participants responded by pressing the direction button (i.e., up, down, left, or right) on the response box indicating the side of the computer monitor on which each image was originally displayed (e.g., press UP if the image was originally shown on the topside of the monitor).

##### Delayed Recognition Memory (DRM)

The DRM task was the follow-up to the IRM task and therefore comprised only a memory recognition phase. Participants were shown a sequential series of 30 images; 15 of these images matched those shown during the encoding phase of the IRM task, with the remaining 15 images being new. Participants pressed the ‘yes’ button press on the response box if the current image was one that they were shown during the memory encoding phase of the IRM task. Alternatively, participants pressed the ‘no’ button if they had not seen that image before.

The primary outcome for this trial was response time (ms) for each SUCCAB task, while response accuracy (%) for each task was treated as a secondary outcome. To control for a response speed–accuracy trade-off [42], a performance score (secondary outcome) for each SUCCAB task (except SRT) was also calculated by dividing response accuracy (%) by mean response time (ms) with a higher score indicating better performance [40,43].

### 2.4. Cardiovascular Assessments

Cardiovascular function was assessed using the SphygmoCor device (Model XCEL, AtCor Medical, Sydney, Australia). The device provides an assessment of conventional brachial systolic and diastolic blood pressures. An appropriately sized brachial cuff was applied to the participant’s right arm whilst in the supine position (following 5-min rest in a quite airconditioned room). The XCEL device was programed to perform three sequential assessments (one-minute interval between assessments), with the result being the mean of the second and third assessments. Following these assessments, the device completed a fourth partial inflation to record the brachial waveform, after which a mathematical transfer function was automatically applied by the device to derive the aortic waveform, from which the XCEL device determines aortic systolic and diastolic blood pressures. Brachial and aortic pulse pressures were calculated by subtracting diastolic from systolic pressure, while mean arterial pressure was calculated using the equation (2 x diastolic + systolic)/3 [44]. The XCEL system also provides several measures of arterial stiffness, including augmentation pressure and the augmentation index. The augmentation pressure and the augmentation index are indices of peripheral arterial stiffness (we report augmentation index normalized to a heart rate of 75 beats per minute; AiX@75).

The XCEL device was also used to assess carotid–femoral pulse wave velocity (CF-PWV), considered to be the gold-standard for assessing aortic stiffness [45]. Briefly, an inflatable pressure cuff (such as the brachial cuff above) was applied to the upper region of the participant’s right thigh. Upon inflation, this cuff detected and recorded the participant’s femoral pressure waveform, whilst a pen-like tonometer (pressure sensor) was applied to the participant’s right carotid artery. The CF-PWV (meters per second) is automatically calculated by the XCEL device via dividing the distance (in millimeters) between the carotid and femoral measurement sites by the pulse transit time (i.e., the time it takes for the pulse to travel from the carotid artery to the femoral artery) in milliseconds. A higher CF-PWV reflects increased aortic stiffness.

### 2.5. Blood Fatty Acid Concentration

**Figure 2:**
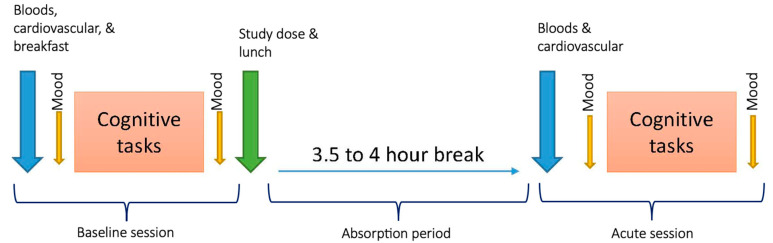
Overview of study procedures on experimental testing days.

Participants provided two blood samples (venepuncture was performed by the research nurse working on the trial) on each of the two experimental testing days. The first sample was collected following an overnight fast, prior to the commencement of pre-dose testing (See Figure 2). The second was a non-fasting sample, occurring 3.5–4-h after the experimental treatment was consumed (participants only consumed the standardized lunch provided and plain water during the 3.5–4-h absorption period).

The procedure for processing blood samples to determine red blood cell (RBC) and plasma fatty acid concentrations has been outlined previously [46]. In brief, each blood sample was collected into a 10 mL ethylenediaminetetraacetic acid (EDTA) tube. The sample was centrifuged (4000 rpm) for five minutes at 4 °C, after which two 1 mL plasma aliquots were extracted into separate cryovials and stored in a −80 °C freezer. The remaining plasma and buffy coat were removed and discarded, while the packed RBC was washed with 0.9% cold saline via a few gentle inversions. The tube was then centrifuged (4000 rpm) for five minutes at 4 °C, with the subsequent supernatant discarded—this was repeated twice more. Finally, two 1 mL aliquots of RBC were extracted into separate cryovials and stored in a −80 °C freezer. The RBC and plasma samples were later dispatched via courier to the South Australia Health and Medical Research Institute (SAHMRI) Fatty Acid Laboratory (Adelaide, Australia) for analysis. Fatty acid concentrations within RBC are reported as a percentage of overall fatty acid content, whereas plasma fatty acids are reported in mg/L plasma. Total plasma ω-3 PUFA, and specifically DHA and EPA concentrations are reported here. The SAHMRI Fatty Acid Laboratory calculated total plasma ω-3 PUFA as the sum of alpha-linolenic, eicosapentaenoic, docosapentaenoic, and docosahexaenoic ω-3 PUFA.

### 2.6. Procedure

Prospective participants were initially screened via telephone to determine eligibility (see Section 2.1). Individuals satisfying initial eligibility criteria were invited to attend our lab at Swinburne University of Technology (Hawthorn, Melbourne, Australia) for an in-person screening/familiarization visit. During this visit, participants provided written informed consent before completing additional screening measures (i.e., mini-mental state examination, and Beck depression inventory 2nd edition) as final confirmation of eligibility. All eligible participants were then familiarized with the study procedures and assessments (e.g., computerized cognitive assessments). The dates on which participants would complete the two experimental testing visits was arranged prior to participants going home. Participants were also provided information on how to prepare for the experimental testing visits. These requirements included consuming a low-fat meal the night prior to the visit (avoiding fish/seafood and other foods high in ω-3 PUFA), while abstaining from alcohol (12 h prior), caffeine (10 h prior), or vigorous physical activity (12 h prior) prior to testing. Participants were instructed to fast from 10 pm the night before their visit.

The first experimental testing visit was scheduled to occur within 14 days of the in-person screening/familiarization visit, while the second experimental testing visit was scheduled to occur 7 days (±3 days) after the first experimental testing visit. All procedures were completed in dedicated testing rooms which were free from outside distraction.

An overview of each experimental testing day is provided in Figure 2. The experimental procedure was identical on both testing days. Upon arrival at our lab (testing began in the morning), participants provided a fasting blood sample prior to cardiovascular function being assessed. Afterwards, participants were provided breakfast—a choice of whole meal toast with jam/vegemite/peanut butter, or cereal. Participants were provided the same breakfast the following testing visit. Afterwards, participants would complete a battery of computerized questionnaires to assess mood (not reported here), followed by completion of the computerized cognitive tasks (i.e., SUCCAB then CDB). Participant mood was assessed again immediately after completing the cognitive tasks. Once testing was complete, participants were provided their treatment (ω-3 PUFA or placebo) mixed into a single serving (140 g) of Greek yogurt (the crossover design meant that participants who had received the placebo during visit 1 would subsequently receive the ω-3 PUFA treatment during visit 2 and vice versa; administration order was randomized and counterbalanced). Participants were required to consume the yogurt within 10 min. Participants were then provided their lunch—a wholemeal roll (including chicken/ham or no protein and any/all of—tomato/carrot/lettuce/onion/cucumber). Participants were provided an identical lunch the following visit. Participants were instructed that they could not consume any other food/drink, except water, or consume any supplements/medications during the subsequent 3.5–4-h absorption break. After the break, participants provided a non-fasting blood test before repeating the testing procedure as described already (alternative versions of the cognitive tasks were used for each testing session). Further, at the end of the second testing day, participants were asked to report which day they thought they received the ‘real’ treatment and provide any reasons informing their opinion. Participants were asked about changes to their health at the start and end of each testing visit as a means of detecting adverse events. Participants were debriefed once the second experimental testing day was complete.

### 2.7. Statistical Analysis

Participant data were initially screened for invalid values, followed by examination for univariate outliers and violations of normality. Group (i.e., treatment) differences in plasma fatty acid concentrations were examined (paired samples *t*-tests) to demonstrate increased total ω-3 PUFA (sum of alpha-linolenic, EPA, docosapentaenoic, and DHA ω-3 PUFA) and specifically DHA and EPA concentrations following ingestion of Driphorm^®^ HiDHA^®^ 360. Conversely, treatment effects for primary (cognition—response time) and secondary (i.e., cognition—response accuracy and performance score; cardiovascular function) outcomes were examined using a two-way repeated measures Analysis of Variance (ANOVA), with all assumptions for this statistical model being satisfied. Treatment by time interaction effects are reported, with the results of pairwise comparisons reported when ANOVA interaction effects are significant. Statistical significance was set at *p* < 0.05. All analyses were performed using IBM SPSS Statistics version 28 (IBM Corp., Armonk, NY, USA).

## 3. Results

### 3.1. Participant Demographics

For an overview of participant demographics, see Table 1. Most (*n* = 27) participants had completed tertiary education and were currently in full-time employment. All participants demonstrated normal global cognitive functioning according to MMSE performance.

Most participants were overweight (BMI, 25.0 < x < 30.0 kg/m^2^; *n* = 16), with nearly equal numbers having a healthy weight (BMI, 18.5 < x < 25.0 kg/m^2^, *n* = 6) or living with obesity (BMI, ≥30.0 kg/m^2^, *n* = 7). Seven participants had stage-one hypertension, while one participant had stage two hypertension according to current American Heart Association guidelines [47]. The remaining 21 participants had normal (*n* = 16) or ‘elevated normal’ (*n* = 5) blood pressure.

The mean RBC ω-3 Index of the trial sample was 5.48% (*n* = 23). Based on the mean ω-3 index, the sample may be characterized as having an ‘intermediate’ risk of coronary heart disease, as per references published in Harris and von Schacky [48].

### 3.2. Primary Analyses

There was no significant treatment by time interaction effect on mean response time for any of the SUCCAB tasks. The removal of significant outliers or the transformation of skewed data did not change the results.

### 3.3. Secondary Analyses

#### 3.3.1. SUCCAB Response Accuracy and Performance Scores

As shown in Table 2, there was no significant treatment by time interaction effect upon mean response accuracy or performance score for any of the SUCCAB tasks. The removal of significant outliers or the transformation of skewed data did not change these results.

#### 3.3.2. Cardiovascular Function

A total of 28 participants had complete pre-dose and post-dose blood pressure (brachial and aortic) and peripheral arterial stiffness data, while complete CF-PWV data were only available for 26 participants. Estimated marginal means, summarizing participant cardiovascular function, are provided in Table 3.

A significant treatment by time interaction effect was observed for aortic systolic blood pressure (aSBP; Table 3). Pairwise comparisons reveal that there was no significant difference in aSBP (i.e., ω-3 PUFA versus placebo) either before (mean difference = 1.07 mmHg, *p* = 0.364, 95% CI: -1.31, 3.45) or after consuming the treatments (mean difference = -1.64 mmHg, *p* = 0.086, 95% CI: -3.53, 0.25). However, mean aSBP significantly declined between the pre-dose and post-dose assessment on the day participants consumed the ω-3 PUFA (DHA + EPA) treatment (mean difference = −4.11 mmHg, *p* = 0.004, 95% CI: −6.75, −1.47), but not following consumption of the placebo (mean difference = −1.39 mmHg, *p* = 0.122, 95% CI: −3.19, 0.04).

There was a significant treatment by time interaction effect on aortic mean arterial pressure (aMAP; Table 3). Pairwise comparisons reveal no significant difference in aMAP (i.e., ω-3 PUFA versus placebo) before (mean difference = 0.21 mmHg, *p* = 0.793, 95% CI: −1.45, 1.88) or after consuming treatments (mean difference = −1.45 mmHg, *p* = 0.099, 95% CI: −3.20, 0.29). Mean aMAP significantly declined between the pre-dose and post-dose assessment on the day participants consumed the ω-3 PUFA (DHA + EPA) treatment (mean difference = 3.54 mmHg, *p* = 0.002, 95% CI: −5.59, −1.48) but also after consuming the placebo (mean difference = −1.87 mmHg, *p* = 0.011, 95% CI: −3.28, −0.46). No other significant differential treatment effects for cardiovascular function were observed.

#### 3.3.3. Plasma ω-3 PUFA Concentrations

**Figure 3:**
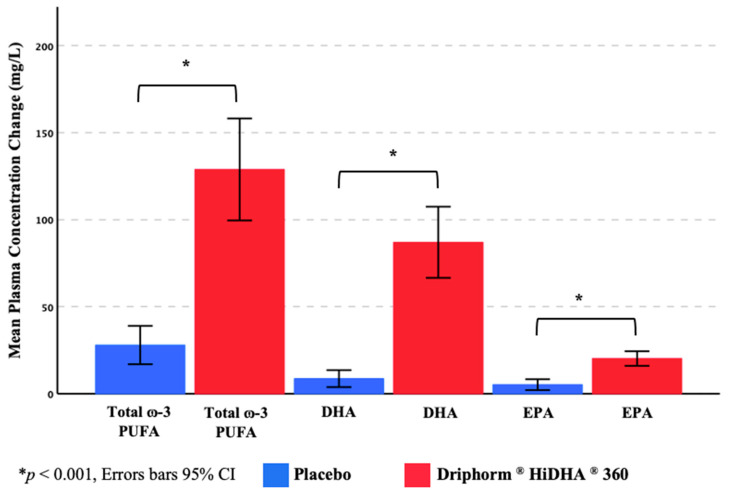
Change in plasma ω-3 PUFA concentrations.

Twenty-two participants had complete plasma fatty acid data. Mean pre-dose (i.e., fasting) plasma concentrations (mg/L; Table 3) of total ω-3 PUFA (calculated as combined total of alpha-linolenic acid, EPA, docosapentaenoic acid, and DHA), DHA, and EPA on each testing day were comparable (Total ω-3 PUFA, t(21) = 1.63, *p* = 0.119; DHA, t(21) = 1.79, *p* = 0.088; EPA, t(21) = 2.02, *p* = 0.057). However, the pre-dose-to-post-dose change in these measures was significantly different between the two treatments, with a markedly greater change apparent following consumption of the ω-3 PUFA (DHA + EPA) treatment relative to placebo (Figure 3).

### 3.4. Efficacy of Treatment Blinding

Following the completion of the final experimental visit, participants were asked to estimate when they had received the high dose ω-3 PUFA (DHA + EPA) treatment. While 25 of 29 participants correctly identified when they received the active treatment, less than half of the sample based their estimate on taste (*n* = 8), or taste in conjunction with texture (*n* = 3) or a subjective psychological change (*n* = 1). Interestingly, 13 participants (45%) based their estimate solely on a subjective psychological effect (e.g., improved subjective mood across the day, self-perceived cognitive benefits), though only 11 were correct in their estimation.

### 3.5. Safety and Tolerance of Treatments

A total of 58 health reports were made by participants during the trial, of which 25 were deemed to be adverse events (AE), and there was 1 suspected adverse reaction (AR). Only one AE was possibly linked to the placebo (i.e., gassiness) and another linked to the ω-3 PUFA (DHA + EPA) treatment (i.e., mild diarrhea), though the participant believed it to be due to the lunch provided. One AR (mild headache of approximately 30 min duration) was considered to be potentially linked to the active treatment as the participant experienced this only after receiving the ω-3 PUFA (DHA + EPA) treatment, though it is also possible this could have occurred as a result of the rigor of testing on the day. Overall, the high dose of ω-3 PUFA (DHA + EPA) delivered by Driphorm^®^ HiDHA^®^ 360 appears to have been well tolerated.

## 4. Discussion

The present trial aimed to examine whether a single high dose of ω-3 PUFA (DHA + EPA), incorporated into a meal (i.e., a single serving of Greek yogurt), would provide acute benefits to cognitive performance in middle-aged adults. Compared to placebo, the high dose of ω-3 PUFA (DHA + EPA) provided no acute benefit to any of the cognitive outcomes. However, the ω-3 PUFA (DHA + EPA) treatment did have an acute effect on cardiovascular function by lowering aSBP (pre-dose to post-dose), an effect which was not apparent following consumption of the placebo.

While acute improvements in blood pressure (i.e., aSBP) were demonstrated, most participants in our sample had relatively healthy blood pressures to begin with. It may be that the reductions in systolic blood pressure we observed were insufficient to induce any acute benefits to cognitive function. However, it is also possible that acute changes in blood pressure are less predictive of cognitive function, compared to acute alterations to endothelial function or cerebrovascular reactivity. In their review, Bell and colleagues [23] indicate that changes in endothelial function (i.e., vasodilation and enhanced blood flow) as well as nitic oxide synthesis are likely mechanisms facilitating acute cognitive effects following flavonoid intake. More recently, Gratton and colleagues [26] determined that adults demonstrating acute improvements in cognitive performance (i.e., incongruent Stroop) following a high dose of cocoa flavanols were also those who experienced enhanced cerebrovascular reactivity (using fNIRS) during a hypercapnia challenge. Considering studies have also reported acute benefits to endothelial function and vascular reactivity following ω-3 PUFA ingestion [29,30], a similar effect may have occurred here; however, without having assessed endothelial function (e.g., flow mediated dilation), we could not determine whether cognitive effects are apparent when differentiating endothelial function ‘responders’ and ‘non-responders’. The use of carotid doppler and/or fNIRS could occur in future replications to explore whether changes in cognitive performance are dependent upon changes in endothelial and cerebrovascular reactivity following ω-3 PUFA (DHA + EPA) intake.

We observed a significant decline in aSBP on the day participants consumed the high dose of ω-3 PUFA (DHA + EPA), but not placebo, though the difference between the two treatments during the post-dose assessment only approached significance. The significant change in aSBP over time (i.e., pre-dose to post-dose) is consistent with earlier studies reporting improved blood pressures following chronic ω-3 PUFA intake [49], though we did not observe additional effects upon vascular tone (i.e., arterial stiffness). Although elevated tissue membrane ω-3 PUFA content may be an important predictor of reduced blood pressure following chronic ω-3 PUFA supplementation [50], the current study indicates that acute reductions in blood pressure are also possible and that these may be driven by more immediate changes in blood plasma ω-3 PUFA concentrations. It is possible that increased plasma ω-3 PUFA, specifically DHA and EPA, facilitates acute improvements in blood pressure via elevating nitric oxide production. In fact, post-prandial increases in total nitrite levels (which index endothelial nitric oxide formation) have been reported four hours after ingestion of ω-3 PUFA [30]. However, as we did not assess nitric oxide levels in this study, this mechanism is speculative and could be explored in a future replication study.

The current study has several strengths. First, the use of an advanced cardiovascular assessment monitor (i.e., SphygmoCor XCEL) allowed aortic blood pressures to be assessed. In this study, aortic blood pressure, specifically systolic blood pressure, appeared to be more sensitive to acute effects of high-dose ω-3 PUFA (DHA + EPA) than brachial systolic blood pressure. Differential changes in systolic blood pressure would have been missed if we only assessed brachial pressures using a conventional blood pressure monitor. Second, high compliance with treatment consumption was apparent in the current study, and the treatment appeared to be well tolerated subsequently minimizing dropout. Nonetheless, the trial also has some limitations. Firstly, the trial only included male participants, which prevents the generalization of potential effects to middle-aged females. Future studies should explore the acute effects of a high-dose ω-3 PUFA (DHA + EPA) in a larger sample, incorporating an equal number of males and females. A second limitation is that endothelial function was not assessed (e.g., flow mediated dilation). It may be that cognitive effects are more likely to occur in endothelial ‘responders’ than ‘non-responders’. We had initially planned on utilizing carotid doppler in this trial. However, equipment failure at study outset prevented us from collecting such data. Another possible limitation of the current study is that both treatments may have included components potentially capable of lowering blood pressure (aside from ω-3 PUFA). Both treatments contain milk protein, specifically sodium caseinate (dose not indicated). Milk proteins including casein (sodium caseinate or calcium caseinate) have been shown to facilitate reduced blood pressures [51,52] and improved endothelial function [53]. Milk proteins such as casein and their relevant peptides facilitate cardiovascular effects via direct (e.g., inhibition of angiotensin converting enzyme) and indirect pathways [54]. It is therefore possible that the effects on blood pressure observed are due to ω-3 PUFA (DHA + EPA) in combination with milk proteins such as casein. However, the extent to which milk proteins may facilitate acute effects on blood pressure is relatively uncertain. We are aware of only one study exploring the post-prandial effects of whey (45 g) or sodium caseinate (45 g) on cardiovascular function, which reported null effects upon brachial blood pressure in a sample of middle-aged overweight/obese postmenopausal females [55].

Future studies should aim to test whether a single high dose of ω-3 PUFA (DHA + EPA), incorporated into a meal, provides acute benefits to cognitive performance in a larger sample of middle-aged males and females. The extent to which acute cognitive changes are influenced by acute alterations to endothelial function or cerebrovascular reactivity following ω-3 PUFA (DHA + EPA) intake should be explored in a sample specifically selected for clinically confirmed hypertension. It is also recommended that future studies explore possible biochemical mechanisms determining the potential effects of high dose ω-3 PUFA (DHA + EPA) on cognitive and cardiovascular function.

## 5. Conclusions

The present study demonstrated that a single high dose of ω-3 PUFA (DHA + EPA), incorporated into a meal, may acutely improve blood pressure in healthy middle-aged males. Contrary to our expectations, there were no improvements in any of the cognitive outcomes following the single high dose of ω-3 PUFA (DHA + EPA). Further work is required to assess whether acute benefits are attainable for cognitive performance.

## Figures and Tables

**Table 1:** Demographic characteristics.

	M (%)	SD	Range
Age (years)	52.79	5.29	42.00–60.00
Education (years)	16.78	2.19	11.00–21.00
Highest education achieved, *n* (%)			
Secondary	2 (6.90)		
Tertiary	19 (65.50)		
Postgraduate	8 (27.60)		
Current employment status, *n* (%)			
Student	1 (3.40)		
Part time/casual	3 (10.30)		
Full time	18 (62.10)		
Retired	4 (13.80)		
Unemployed	3 (10.30)		
Height (cm)	178.03	7.13	162.20–194.00
Body weight (kg)	87.12	14.21	66.50–130.70
Body mass index (kg/m^2^)	27.44	3.79	21.10–38.60
Systolic blood pressure (mmHg) *	127.17	9.46	112–153
Diastolic blood pressure (mmHg) *	77.86	9.74	43–90
RBC ω-3 PUFA Content (as %)			
Total ω-3 PUFA ^†^	8.51	1.52	4.33–10.71
DHA	4.54	1.17	2.13–6.68
EPA	0.94	0.24	0.47–1.43
ω-3 Index	5.48	1.29	2.60–7.82

* Brachial blood pressures were assessed during the screening/familiarization visit. ^†^ Total ω-3 PUFA calculated as sum of RBC alpha-linolenic, EPA, docosapentaenoic, and DHA ω-3 PUFA. Abbreviations: RBC, red blood cell; ω-3, omega-3; PUFA, polyunsaturated fatty acid; DHA, docosahexaenoic acid; EPA, eicosapentaenoic acid.

**Table 2:** Estimated marginal means for SUCCAB response time, response accuracy, and performance scores.

	Placebo				ω-3 PUFA *				Interaction ^1^	
SUCCAB Outcome	Pre-Dose		Post-Dose		Pre-Dose		Post-Dose			
	M	SD	M	SD	M	SD	M	SD	F	*p*
Response Time (ms)										
SRT	267.56	30.60	274.59	38.95	270.82	32.18	277.99	36.50	<0.01	0.982
CRT	462.59	73.54	457.77	71.82	468.71	61.31	459.35	75.26	0.19	0.667
IRM	1085.31	276.22	962.64	155.89	1106.11	220.71	979.62	176.49	0.01	0.929
CS	726.32	88.65	674.45	110.13	706.73	85.40	668.10	89.76	0.87	0.359
ICS	862.97	131.10	808.63	111.05	863.14	158.62	797.14	145.71	0.31	0.584
Stroop Effect	136.65	89.64	134.18	92.32	156.41	107.33	129.04	92.85	1.29	0.267
SWM	946.34	258.96	912.45	192.60	996.82	296.02	926.34	200.52	0.65	0.426
CRM	1024.55	219.03	1007.73	217.05	1063.74	274.45	1008.89	196.42	1.19	0.285
DRM	1087.32	190.06	1089.76	190.96	1124.48	283.93	1108.86	213.40	0.14	0.710
Response Accuracy (%)										
CRT	98.28	2.76	96.72	4.07	97.93	2.84	98.10	2.81	2.64	0.115
IRM	84.25	8.54	85.98	7.89	82.41	9.96	84.83	9.28	0.10	0.756
CS	99.14	1.67	98.70	2.19	98.71	1.72	98.62	2.07	0.23	0.634
ICS	98.19	2.30	98.02	3.50	98.45	2.35	97.33	2.49	1.14	0.296
SWM	85.45	10.71	85.90	9.11	86.02	9.33	84.46	10.33	1.18	0.287
CRW	81.38	20.04	86.03	12.42	86.90	13.12	86.03	12.91	2.04	0.164
DRM	72.53	12.07	73.47	9.93	77.20	11.81	74.53	13.67	1.58	0.221
Performance Score										
CRT	217.24	32.23	215.59	30.28	212.32	28.16	217.92	29.20	2.39	0.133
IRM	81.48	18.64	91.19	15.04	77.31	17.75	89.04	17.48	0.38	0.545
CS	138.64	18.81	149.50	20.65	141.61	16.98	150.19	20.42	0.61	0.440
ICS	116.48	18.82	123.60	18.65	117.72	21.24	125.78	21.38	0.08	0.775
SWM	97.12	28.70	98.89	26.78	93.41	27.54	96.00	26.85	0.03	0.856
CRW	83.34	27.05	89.07	23.12	86.15	23.21	88.32	21.44	0.87	0.358
DRM	68.65	15.97	69.02	13.36	72.08	17.91	69.27	17.21	0.88	0.357

Notes: ^1^ Treatment by time interaction using two-way repeated measures ANOVA. For all outcomes, *n* = 29, except for DRM where *n* = 25 (four participants response accuracy < 50%, i.e., less than chance, and were therefore excluded). * Treatment was Driphorm^®^ HiDHA^®^ 360 and contained DHA + EPA ω-3 PUFA. Abbreviations: SRT, simple reaction time; CRT, choice reaction time; IRM, immediate recognition memory; CS, congruent stroop; ICS, incongruent stoop; SWM, spatial working memory; CRM, contextual recognition memory; DRM, delayed recognition memory.

**Table 3:** Estimated marginal means for participant cardiovascular function and concentration of plasma ω-3 PUFA.

	Placebo				ω-3 PUFA *				Interaction ^1^	
Outcome	Pre-Dose		Post-Dose		Pre-Dose		Post-Dose			
	M	SD	M	SD	M	SD	M	SD	F	*p*
Brachial Pressures (mmHg)										
SBP	115.18	10.86	113.82	8.97	115.57	10.33	111.79	7.27	3.25	0.082
DBP	71.89	6.78	69.71	6.53	71.50	6.45	68.18	4.57	1.48	0.235
PP	43.29	6.42	44.11	5.32	44.07	5.54	43.61	5.97	0.72	0.404
MAP	86.32	7.79	84.42	7.00	86.19	7.52	82.71	4.86	3.61	0.068
Aortic Pressures (mmHg)										
SBP	105.89	9.60	104.50	8.20	106.96	9.95	102.86	6.52	5.95	**0.022**
DBP	72.68	6.79	70.57	6.59	72.46	6.61	69.21	4.57	1.57	0.221
PP	33.21	4.79	34.50	4.76	33.93	4.59	33.64	4.51	1.77	0.195
MAP	83.75	7.51	81.88	6.83	83.96	7.56	80.43	4.86	4.23	**0.050**
Arterial Stiffness										
AP (mmHg)	7.75	3.22	7.57	3.69	8.50	4.41	8.25	3.98	0.01	0.931
AiX@75 (%)	14.50	10.33	14.50	12.49	15.14	12.71	16.04	12.46	0.17	0.682
CF-PWV (m/s)	10.00	0.99	9.86	0.95	9.77	0.89	9.65	0.73	0.01	0.928
Plasma ω-3 Concentration										
Total ω-3 PUFA ^†^ (mg/L)	135.59	37.78	158.40	49.49	126.41	29.48	263.72	86.34	-	-
DHA (mg/L)	59.93	16.51	65.70	19.34	56.18	15.66	150.32	52.39	-	-
EPA (mg/L)	30.69	12.12	34.44	16.56	26.71	8.39	48.32	12.62	-	-

Note: ^1^ Treatment by time interaction using two-way repeated measures ANOVA. For all blood pressure measures, *n* = 28, whereas for CF-PWV, *n* = 26. All plasma ω-3 PUFA concentrations, *n* = 22. * Treatment was Driphorm^®^ HiDHA^®^ 360 containing DHA + EPA ω-3 PUFA. ^†^ Total ω-3 PUFA calculated as sum of plasma alpha-linolenic, EPA, docosapentaenoic, and DHA ω-3 PUFA. Abbreviations: SBP, systolic blood pressure; DBP, diastolic blood pressure; PP, pulse pressure; MAP, mean arterial blood pressure; AP, augmentation pressure (a measure of peripheral arterial stiffness); AiX@75, augmentation index—normalized for heart rate of 75 beats per minute (a measure of peripheral arterial stiffness); CF-PWV, carotid femoral pulse wave velocity (a measure of central arterial stiffness); ω-3, omega-3; *PUFA*, polyunsaturated fatty acid; DHA, docosahexaenoic acid; EPA, eicosapentaenoic acid. Statistically significant (*p* < 0.05) results in bold.

## Data Availability

The data presented in this study is not publicly available due to privacy and ethical restrictions. Requests for data by suitably qualified individuals may be sent to the corresponding author.
